# Diagnosis and Management of Cesarean Scar Ectopic Pregnancy: A Case Study

**DOI:** 10.7759/cureus.45160

**Published:** 2023-09-13

**Authors:** Sabine Itani, Mohamad Jabin, Harshith Dasara, Koleton Forehand, Gregory DePrisco

**Affiliations:** 1 Medicine, Texas A&M College of Medicine, Bryan, USA; 2 Radiology, Baylor University Medical Center, Dallas, USA; 3 Diagnostic Radiology, Baylor University Medical Center, Dallas, USA

**Keywords:** mri, ultrasonography, ectopic, pregnancy, cesarean section

## Abstract

A cesarean scar ectopic pregnancy (CSEP) designates an ectopic pregnancy within the myometrium of a past uterine incision. Early diagnosis through transvaginal ultrasonography is crucial as an untreated CSEP can lead to serious complications, including hemorrhage, loss of future fertility, and maternal death. We present a case of a 33-year-old female with five previous cesarean sections, who presented at seven weeks of gestation with concerns of a CSEP. Here, we highlight the importance of early diagnosis and maintaining high clinical suspicion in women with multiple previous cesarean sections who present with menstrual abnormalities. The CSEP is a serious condition and requires a high index of suspicion during diagnosis and follow-up. Ultrasound scanning and training should be readily available to quickly identify and treat this life-threatening condition.

## Introduction

A cesarean scar ectopic pregnancy (CSEP) refers to a progressing pregnancy implanted within the myometrium of a previous incision site. The prevalence of this condition has significantly increased over the past few decades, reaching approximately 21% worldwide [1]. With this rise and the advancements in sonographic imaging technology, there has been an increase in the identification of CSEPs, with a prevalence of 1 in every 2,000 pregnancies in the United States [2]. Although the incidence of CSEPs is high, they remain underdiagnosed. At the beginning of the first trimester, the CSEP is usually found incidentally with ultrasonography in asymptomatic women. In some cases, symptoms may include first-trimester pelvic pain and vaginal bleeding. The diagnostic of choice is transvaginal ultrasound (TVUS). In equivocal cases, magnetic resonance imaging (MRI) may be used to confirm the diagnosis. If left untreated, a CSEP can manifest as hemorrhage, loss of future fertility, and possible maternal death [3]. The swift recognition and intervention of this condition are of paramount significance for healthcare practitioners.

## Case presentation

**Figure 1:**
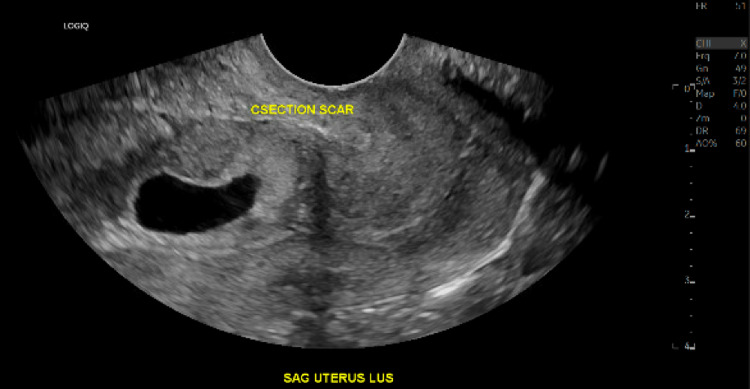
Transvaginal grayscale ultrasound image demonstrating an intrauterine gestational sac in the lower uterine segment at the level of the cesarean section scar.

**Figure 2:**
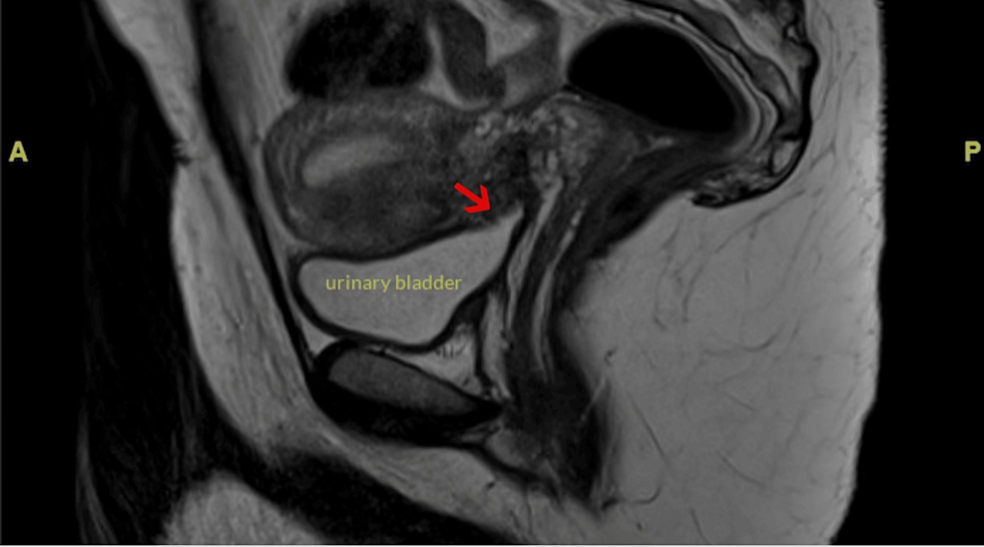
Midline sagittal T2 weighted magnetic resonance image through the pelvis demonstrating chorionic tissue extending beyond the serosal margin of the myometrium and being inseparable from the serosal margin of the urinary bladder dome (red arrow).

A 33-year-old female (G6P5) at seven weeks of gestation presented as a high-risk referral from another facility concerning a CSEP with gestational sac invasion into the myometrium and possibly the bladder. She reported having spotting intermittently for the last several weeks but otherwise described doing very well. She denied any complaints of cramping, abdominal pain, nausea, or vomiting. She has a history of five cesarean sections, which led to concern for serosal involvement at the bladder dome. She presented at the original facility with severe tooth and jaw pain, painless vaginal spotting, and no pelvic or abdominal pain. On physical exam, her left lower molar appeared cracked. Her abdomen was non-tender to palpation and her pelvic exam was unremarkable. Her vital signs were stable. Her laboratory results demonstrated mild microcytic anemia, Rhesus factor positive, and a hCG level of over 24,000. Ultrasound imaging demonstrated an intrauterine gestational sac in the lower uterine segment at the level of the cesarean section scar with a mean sac diameter of 1.3 centimeters (Figure 1). There was no evidence of subchorionic hematoma, pelvic hemorrhage, or uterine perforation. The right ovary appeared normal, while the left ovary contained a 1.5-centimeter corpus luteum cyst. Because placenta accreta could not be excluded by ultrasound, MRI was performed, which revealed chorionic tissue extending beyond the serosal margin of the myometrium and being inseparable from the serosal margin of the urinary bladder dome, raising concern for placenta percreta with the involvement of the serosal bladder (Figure 2). The patient was suspected of being a nonsurgical management candidate but was admitted overnight for a maternal-fetal medicine consultation the following day for confirmation. The consulting physician agreed with the assessment and recommended methotrexate treatment with a repeat ultrasound in 1-2 weeks. The patient was then assigned close outpatient follow-up to ensure a successful resolution and discharged home in good condition later that day.

## Discussion

CSEPs are caused by trophoblastic implantation in the scar tissue due to impaired healing. Most patients are asymptomatic, while some may present with abdominal pain and vaginal bleeding in the first trimester. CSEPs are rare, compromising less than 1% of all pregnancies [1]. However, in recent years, the occurrence has risen due to the escalating prevalence of ectopic pregnancies.

The primary method for diagnosing a suspected CSEP involves the use of combined transvaginal grayscale and color Doppler ultrasonography [4], which is also the most effective approach, exhibiting a sensitivity of 86.4% [5]. The ultrasound criteria employed to identify a CSEP consists of an empty uterus displaying a well-defined endometrium, an unoccupied cervical canal, the presence of a gestational sac implanted in the lower anterior part of the uterine segment, and a thin layer of myometrial tissue situated between the gestational sac and the urinary bladder [3,6].

Diagnosis of a CSEP is established when the uterine cavity and cervical canal are devoid of content, and the gestational sac is located in the anterior section of the uterine isthmus [5]. Moreover, the myometrial thickness at the implantation site is considered atypical if it measures below eight millimeters [7]. This unusual implantation transpires when the blastocyst embeds itself within the scar tissue left by a previous cesarean incision, with an elevated likelihood of such abnormal implantation occurring in women who have undergone multiple cesarean deliveries.

Classified into three discernible types, CSEP's categorization depends on how the gestational sac is positioned relative to the uterine cavity and serosa. These types encompass: CSEP implanted within the niche and extending into the uterine cavity, CSEP located within the myometrium, and CSEP extending toward the urinary bladder and crossing the serosal boundaries [3,8]. While MRI is recognized as a valuable supplementary tool to transvaginal ultrasound and can confirm the diagnosis, it should not serve as the primary diagnostic method in these scenarios.

The identification of the CSEP should take place prior to the ninth week of gestation in order to differentiate between CSEP, cervical pregnancy, and an ongoing spontaneous abortion [4,9]. In this instance, the CSEP was detected at the seventh week of gestation, and the absence of defining characteristics, such as the gestational sac being implanted within the cervix's endocervical canal instead of the front lower uterine segment, eliminated the possibility of a cervical pregnancy [3]. There was no observation of a fetal pole with embryonic cardiac activity, and her cervical os remained closed, thereby ruling out the occurrence of an ongoing spontaneous abortion as well.

Currently, there are several acceptable methods of treatment for CSEPs, which are dependent on clinical presentation, gestational age, and CSEP location [3]. If a patient consents to terminate the pregnancy, medical and/or surgical management must be initiated once the diagnosis is confirmed. Medical management includes injections of methotrexate, while surgical management consists of laparoscopic resection and vacuum aspiration [10]. The goals of both medical and surgical therapy are the same; however, laparoscopic resection is usually reserved until after methotrexate therapy fails.

## Conclusions

The CSEP is a life-threatening gynecological condition that must be diagnosed and treated early in the first trimester. Presenting as a diagnostic dilemma, it necessitates practitioners to uphold a heightened sense of awareness while interpreting images and conducting subsequent evaluations. This study will fill out the gap present.
